# HNF1A：From Monogenic Diabetes to Type 2 Diabetes and Gestational Diabetes Mellitus

**DOI:** 10.3389/fendo.2022.829565

**Published:** 2022-03-01

**Authors:** Li-Mei Li, Bei-Ge Jiang, Liang-Liang Sun

**Affiliations:** ^1^ Research Center for Translational Medicine, Key Laboratory of Arrhythmias of the Ministry of Education of China, Shanghai East Hospital, Tongji University School of Medicine, Shanghai, China; ^2^ Third Department of Hepatic Surgery, Eastern Hepatobiliary Surgery Hospital, Naval Medical University, Shanghai, China; ^3^ Department of Endocrinology and Metabolism, Changzheng Hospital, Naval Medical University, Shanghai, China

**Keywords:** HNF1α, polymorphism, heterogeneity, MODY3, type 2 diabetes, gestational diabetes mellitus

## Abstract

Diabetes, a disease characterized by hyperglycemia, has a serious impact on the lives and families of patients as well as on society. Diabetes is a group of highly heterogeneous metabolic diseases that can be classified as type 1 diabetes (T1D), type 2 diabetes (T2D), gestational diabetes mellitus (GDM), or other according to the etiology. The clinical manifestations are more or less similar among the different types of diabetes, and each type is highly heterogeneous due to different pathogenic factors. Therefore, distinguishing between various types of diabetes and defining their subtypes are major challenges hindering the precise treatment of the disease. T2D is the main type of diabetes in humans as well as the most heterogeneous. Fortunately, some studies have shown that variants of certain genes involved in monogenic diabetes also increase the risk of T2D. We hope this finding will enable breakthroughs regarding the pathogenesis of T2D and facilitate personalized treatment of the disease by exploring the function of the signal genes involved. Hepatocyte nuclear factor 1 homeobox A (HNF1α) is widely expressed in pancreatic β cells, the liver, the intestines, and other organs. HNF1α is highly polymorphic, but lacks a mutation hot spot. Mutations can be found at any site of the gene. Some single nucleotide polymorphisms (SNPs) cause maturity-onset diabetes of the young type 3 (MODY3) while some others do not cause MODY3 but increase the susceptibility to T2D or GDM. The phenotypes of MODY3 caused by different SNPs also differ. MODY3 is among the most common types of MODY, which is a form of monogenic diabetes mellitus caused by a single gene mutation. Both T2D and GDM are multifactorial diseases caused by both genetic and environmental factors. Different types of diabetes mellitus have different clinical phenotypes and treatments. This review focuses on HNF1α gene polymorphisms, HNF1A-MODY3, HNF1A-associated T2D and GDM, and the related pathogenesis and treatment methods. We hope this review will provide a valuable reference for the precise and individualized treatment of diabetes caused by abnormal HNF1α by summarizing the clinical heterogeneity of blood glucose abnormalities caused by HNF1α mutation.

## Introduction

According to the International Diabetes Federation (IDF), there were approximately 463 million adults with diabetes worldwide in 2019. The incidence is increasing, and this value will reach 700 million in 2045 (1). Diabetes has become a considerable public health problem that substantially impacts society and the families of patients (2). The disease is currently divided into four categories: type 1 diabetes (T1D), type 2 diabetes (T2D), gestational diabetes mellitus (GDM) and other special types of diabetes mellitus (3). The clinical manifestations of the different types of diabetes are similar, which greatly hinders the classification of the disease (4). Moreover, there is heterogeneity in the clinical phenotypes of the disease under the same type, which makes the clinical diagnosis of the diabetes type more difficult (4). Correctly diagnosing the type of diabetes is essential for precise treatment. The high heterogeneity of diabetes blurs the classic distinction between diabetes types. Interpreting the heterogeneity has become a major focus in diabetes research. It is necessary to establish a more accurate classification of diabetes. This will enable the precise and personalized treatment of diabetes, including the provision of more appropriate care, the development of hypoglycemic drugs, and the prevention/treatment of complications.

The most common types, i.e., T1D, T2D, and GDM, are multifactorial syndromes related to various gene effects and environmental factors (5). Two rare types, i.e., neonatal diabetes mellitus (NDM) and maturity-onset diabetes of the young (MODY), are caused by a single gene mutation (5, 6). MODY is a type of monogenic diabetes characterized by early-onset (age of diagnosis is usually before 25), autosomal dominant inheritance, no autoimmune process or insulin resistance, retention of endogenous insulin secretion, and no dependence on insulin (7). It is estimated that MODY accounts for approximately 1-2% of all diabetic cases (7). The pathogenesis of T2D and GDM is unclear. T2D is the most important type of diabetes and accounts for more than 90% of the total number of diabetic patients (3). Studies have shown that some monogenic diabetes genes are involved in T2D, with some variants significantly increasing the risk of T2D (8). T2D and GDM have solid genetic factors. Monogenic diabetes provides a good resource for elucidating the pathogenesis and developing personalized care for T2D and GDM. Substantial progress has been made in the research and development of hypoglycemic drugs targeting monogenic diabetes. This has provided the inspiration to further explore the pathogenesis and develop personalized treatments for T2D by studying the monogenic diabetes genes.

Hepatocyte nuclear factor 1α (HNF1α or HNF1A), also known as the MODY3 gene, is the pathogenic gene for MODY3 (7).Common types of HNF1α mutations cause MODY3. Other mutations do not cause MODY3 but significantly increase the risk of T2D, while others increase the risk of GDM. Hyperglycemia in MODY3 is caused by single-gene abnormalities. Unlike the pathogenesis of MODY, a large number of basic and clinical experiments show that T2D and GDM result from genetic and environmental factors. These two factors interact with each other to promote the occurrence of T2D or GDM. Why do different HNF1α mutations cause different types of diabetes? Answering these questions may improve our understanding of diabetes and advance the precise treatment of diabetes. In this review, we first summarize and discuss the relationship between HNF1α gene polymorphisms, MODY3, T2D, and GDM. We then explore the mechanism of hyperglycemia caused by HNF1α mutation by discussing the role of HNF1α in the islets and liver. Finally, we explore the reason why different HNF1α mutations can lead to different types of diabetes by analyzing the protein structure and function. We hope our review will facilitate a more comprehensive understanding of hyperglycemia caused by HNF1α mutation and will be useful for accurate diagnosis and treatment of diabetes, especially the hyperglycemia caused by HNF1α.

## Polymorphism of HNF1A Gene

Human HNF1α is located at q24.31 on chromosome 12 (9) and is a widely expressed tissue-specific transcription factor (10). In the liver, HNF1α regulates numerous liver-specific genes and participates in the metabolism of glucose, fat, and other substances (11–13). In the pancreas, HNF1α controls many pancreatic-specific genes involved in β-cell maturation, growth, and insulin secretion (14–16). The HNF1α gene has a large number of polymorphisms with no specific mutation hotspots, and 894 variants are listed in the Exome Aggregation Consortium (ExAC) database. In total, 1231 variants can be queried from Genome Aggregation Database (gnomAD) (17). These variants range from the HNF1α promoter to the 3’ untranslated region (UTR), including missense, translocation, nonsense, splice mutation, in-frame amino acid deletion, insertion, duplication, or partial and whole gene deletion (17).

**Figure 1:**
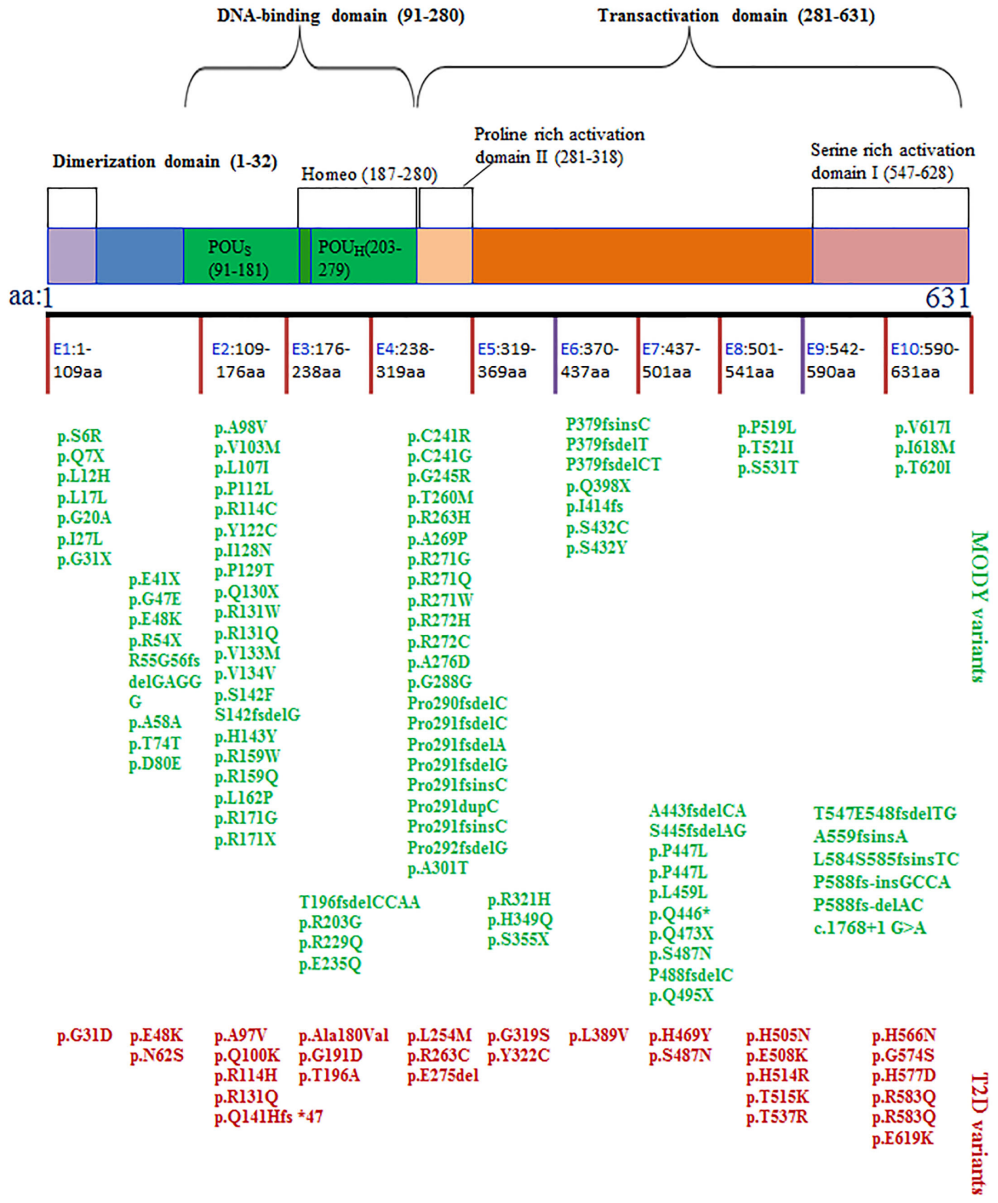
The structure of HNF1α protein and the SNPs associated with MODY3 or T2D. The SNPs in blue often cause MODY3. The SNPs in red often cause T2D.

**Figure 2:**
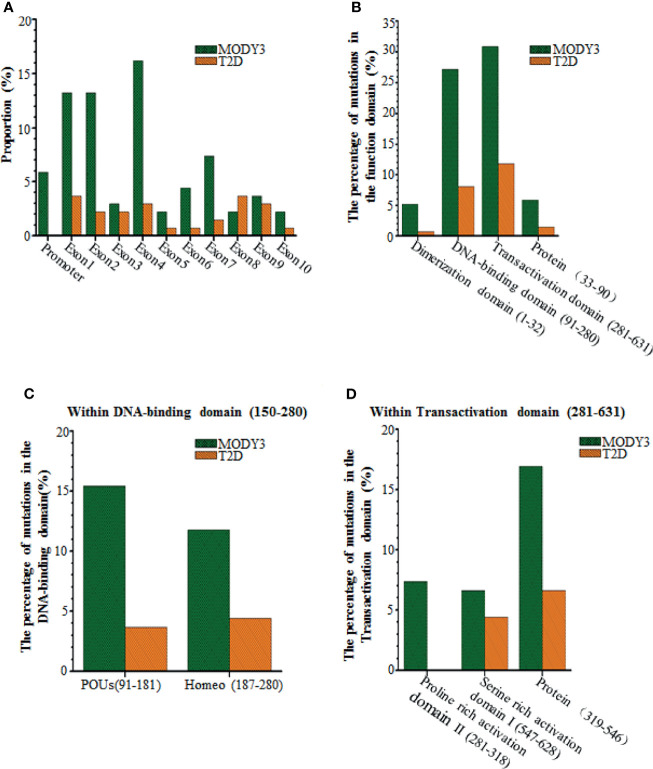
Comparisons of the HNF1a SNPs causing MODY3 with the HNF1a SNPs causing T2D. **(A)** Location within the promoter and 10 exons, **(B)** Location within the functional domains of HNF1α protein, **(C)** Location within DNA-binding domain of HNF1α protein, **(D)** Location within transactivation domain of HNF1α protein.

Although mutations have been observed in all exons, they are most often detected in exons 2 and 4 (**Figures 1**, **2**). Among them, the mutation of exon 4 (p291fsinsc) is the most common (18). In a study of 414 different mutations in 1247 families carrying the HNF1α gene, the most common mutations were missense mutations (55%), frameshifts (22%), splice sites (9%), promoter region mutations (2%) and deletions (1.2%) (19). The mutations caused by these SNPs are as follows: 1) The mutation is located in the exon region and causes the substitution of an amino acid in HNF1α, resulting in a missense mutation (20). 2) The mutation is located in the exon region and causes abnormal shear in the HNF1α transcript (20). For example, although no RNA has been obtained from patients to prove this hypothesis, a synonymous mutation in HNF1α (c.1623 G > A, p.Gln541Gln) involving the last nucleotide of exon 8 was predicted to affect RNA splicing (21). 3) Mutations located in intron regions may generate new splice sites, resulting in pseudoexons (22, 23). 4) The mutation may be located in the promoter region, resulting in reduced gene expression (20).

## Association Between HNF1A Polymorphism and MODY3

HNF1A-MODY3 is characterized as familial diabetes (9, 24). Hyperglycemia usually becomes evident and deteriorates during puberty or early adulthood. Approximately 25% of HNF1A-MODY patients have typical polydipsia, polyuria, polyphagia, and emaciation symptoms in the initial stage, but most patients have none of the above clinical manifestations and only present elevated postprandial blood glucose, usually without ketosis (24, 25). Such patients usually show mild osmotic symptoms (polyuria and polydipsia) or asymptomatic postprandial hyperglycemia, without ketosis or ketoacidosis at age 6-25 (26). However, the clinical symptoms of HNF1A-MODY are very different. The clinical characteristics of HNF1A-MODY differ between and within families, which complicates the diagnostic process. In addition, different MODY3 patients exhibit great heterogeneity in the first-line use of sulfonylureas and the occurrence of complications. Therefore, we hope to provide some valuable information for MODY3 diagnosis, accurate treatment, and early intervention for other carriers of the same mutation in the same family before the occurrence of relevant clinical symptoms by summarizing some significant clinical features of MODY3.

### Clinical Heterogeneity in MODY3

Thus far, hundreds of distinct HNF1α SNPs have been found to cause MODY3 (**Figure 1** and **Supplementary Table 1**). The genetic diversity of MODY3 leads to heterogeneity in the MODY3 clinical phenotype. However, environmental factors also contribute to the clinical heterogeneity of MODY3. The main clinical heterogeneities in MODY3 are listed below.

#### Family History

As a case of autosomal dominant inherited disease, MODY3 patients usually have a family history of diabetes (9, 27, 28). Some studies suggest that the probability of a family history of MODY3 is more than 20 times higher than that of T1D (29). However, some MODY patients may lack a family history of diabetes, possibly due to the following factors (1): the probability of new HNF1A-MODY mutations may be more frequent than expected, and (2) the family members of the patients were not diagnosed because of mild clinical symptoms and signs (29, 30).

#### Age of Onset

Comparing the age of diabetes induced by HNF1α mutation in the literature revealed the age of onset of diabetes was generally 10-16 years (**Table 1**). This finding may be attributed to the high genetic penetrance of the HNF1α mutation and is consistent with previously reported conclusions. It has been reported that 63% of carriers are younger than 25 years, 79% are younger than 35 years, and 96% are younger than 55 years (39). The average age of an HNF1A-MODY diagnosis is 14 years, and the disease is rarely diagnosed in children under 10 years old (40). This finding is consistent with our statistical data (**Table 1**).

**Table 1:** Comparison of the onset ages of diabetes caused by different MODY3 associated SNPs.

Location		Nucleotide Change at DNA Level	Mutation	Age of Onset of the Subject (Yrs, range)	BMI	Ref
Genomical DNA	Codon					
Promoter	Promoter	g.-58A>C	HNF4a binding site	22/23	NA	(31)
Exon 1	47	c.140G>A	p.G47E	12	NA	(32)
Exon 1	48	c.142C>A	p.E48K	12	NA	(33)
Exon 1	54	c.160C>T	p.R54X	14-29	21.6-29.7	(34, 35)
Exon 1	103	c.307G>A	p.V103M	25	23.6	(36)
Exon 1	107	c.319C>G	p.L107I	23.5 ± 5.8 (6/2)	25.3 ± 3.5	(37)
Exon 2	112	c.335C>T	p.P112L	9.9	20.3	(38)
Exon 2	114	c.340C>T	p.R114C	21	22.6	(36)
Exon 2	128	c.383T>C	p.I128N	16	21.2	(27)
Exon 2	131	c.392C>T	p.R131W	10-20	NA	(32)
Exon 2	143	c.427C>T	p.H143Y	7	21.5	(27)
Exon 2	171	c.511C>G	p.R171G	21	18.2	(36)
Exon 2	171	c.511C>T	p.R171X	11-26	NA	(32)
Exon 3	196	c.587_590delCCAA	T196fsdelCCAA	31	NA	(32)
Exon 3	229	c.686G>A	p.R229Q	21-36	NA	(32)
Exon 3	235	c.703G>C	p.E235Q	23	20.8	(36)
Intron 3	Intron	c.714-1G>A	IVS3−1G>A	24	NA	(32)
Exon 4	241	c.721T>G	p.C241G	12	NA	(33)
Exon 4	245	c.733G>C	p.G245R	25	25.5	(36)
Exon 4	263	c.788G>A	p.R263H	17	16.3	(36)
Exon 4	263	c.787C>T	p.R263C	13-27	NA	(32)
Exon 4	271	c.812G>A	p.R271Q	14	16.6	(36)
Exon 4	271	c.811C>T	p.R271W	16	NA	(32)
Exon 4	276	c.827C>A	p.A276D	24	NA	(32)
Exon 4	291	c.873delA	Pro291fsdelA	12	NA	(33)
Exon 4	291	c.8743elC	P291fsinsC	6-54	NA	(32)
Exon 5	349	c.1047C>G	p.H349Q	23	24.2	(36)
Exon 6	379	c.1136-1137delT	P379fsdelT	13	21.4	(27)
Exon 6	379	c.1136-1137delCT	P379fsdelCT	11-20	NA	(32)
Exon 7	445	c.1333_1334delAG	S445fsdelAG	12–13	NA	(32)
Exon 7	447	c.1340C>T	p.P447L	18	22.1	(27)
Exon 7	447	c.1340C>G	p.P447L	17	NA	(32)
Exon 7	487	c.1460G>A	p.S487N	20	18.9	(36)
Exon 8	531	c.1592G>C	p.S531T	35	NA	(32)
Exon 9	559	c.1677^1678insA	A559fsinsA	19	22.9	(27)

NA, Not available.

The location of the mutation determines the age of diagnosis of abnormal blood glucose. As shown in **Supplementary Table 1** and **Table 1**, the SNPs that cause MODY3 are concentrated in exons 1, 2, and 4, with relatively few SNPs in exons 8-10. Bellanne-Chantelot C et al. reported that patients with exon 8-10 mutations were diagnosed with HNF1A-MODY3 8 years later than those with exon 1-6 mutations (41). We found that patients with mutations affecting the dimerization domain or the DNA binding domain had a lower onset age (**Table 1** and **Figure 1**). Patients with truncated mutations had a lower onset age than those with missense mutations. The above findings may indicate that the DNA binding region of HNF1α plays a more important role in regulating blood glucose, and the domain that forms a dimer plays the second most important role.

An individual’s genetic background and environment may also affect the onset age of HNF1A-MODY3. For example, intrauterine exposure (mutation inherited by the mother and hyperglycemia during pregnancy) can lead to an early onset age of less than 12 years (25). There are also differences in the age of onset with the same SNP. For example, a survey of MOD3 in Britain uncovered five diabetes patients in three generations of the same family with the R54X genotype (34, 35). Among them, the proband and his brother, mother, and grandmother were diagnosed with diabetes at puberty, while their uncle carried the same SNP and was only diagnosed with T2D at the age of 29 (24). Another family from China also exhibited a difference in the onset age of diabetes with R54X SNPs (35). The daughter was diagnosed with MODY3 at the age of 19, while the mother was first diagnosed with T2D at the age of 27. Among the above six R54X carriers (**Table 2**), 50% were first diagnosed with diabetes before the age of 25 years, which conforms to the standard age of diagnosis for MODY3. However, 50% of the diagnoses occurred at an age over 25, but all occurred before the age of 30. Therefore, HNF1α may play decisive roles in the development of abnormal blood glucose, but personal living habits, including eating, daily life, physical exercise, and other genetic factors that can cause abnormal metabolism, also play a role. Therefore, although a single HNF1α gene mutation is the cause of MODY3, environmental factors also play a role in its pathogenesis.

**Table 2:** The clinical characteristics of two R54X variants from different countries.

Pedigree	Kindred/Generation Subject	Sex	Relation	Age at Diagnosis	BMI	Treatment	Ref
A family from U.K.	I: 2	F	Grandmather	Adolescence	NA	Insulin	(34)
	II: 1	F	Mother	18	25.2	Insulin	
	II: 2	M	Mother’s brother	29	NA	NA	
	III: 1	M	Proband	14	29.7	Insulin	
	III: 2	M	Brother	17	21.6	Insulin	
B family from China	I: 1	F	Mother	27	NA	sulphonylurea (gliclazide)	(35)
	II: 1	F	Proband	19	22	insulin,glybenclamide	

NA, Not available.

#### Insulin

The basal insulin level and insulin response to corresponding high glucose stimulation differ among patients with HNF1A-MODY3. Byrne MM et al. found that some patients with MODY3 had normal fasting blood glucose, while others had very high fasting blood glucose (42). Compared to those with normal blood glucose, the basal insulin secretion of the MODY3 patients was lower, but their insulin secretion increased in response to high-glucose stimulation; some patients showed a limited change, while others patients showed a decrease (42). This finding may indicate that HNF1α plays a direct role in regulating insulin secretion in islet β cells in response to high glucose or other stimuli. The difference may be a result of different mutations in HNF1α.

#### Glycosuria

Patients with HNF1α mutations may exhibit glycosuria; however, not all HNF1A-MODY patients do. Some studies have found that diabetes accounts for approximately 30-40% of all HNF1A-MODY patients (40, 43). For example, a study involving 11 HNF1A-MODY patients in Japan found that about 36% of HNF1A-diabetic patients experienced renal dysfunction (43). Another survey by Amanda Stride et al. also showed similar results (40). These authors found that 38% of mutation carriers developed glycosuria (40) 2 hours after oral glucose. Glycosuria in MODY3 may be caused by an impaired renal tubular transport of glucose and reduced glucose reabsorption in the proximal renal tubules (40, 44, 45). The renal glucose threshold of glucose reabsorption is low.

The renal glucose threshold of HNF1A-MODY patients is lower than that of healthy individuals with positive urine glucose, likely because HNF1α regulates the expression of the glucose transporter sodium-glucose cotransporter 2 (SGLT2) in the kidney because HNF1α can bind the promoter region of SGLT2 (46). In general, HNF1α needs to form a dimer to regulate the transcriptional expression of its target gene (11, 47). Both HNF1α and HNF1β are the most common transcription factors in the HNF transcription factor family and play important roles in the pancreas, liver, and kidney (10). These factors often form heterodimers (48). It has been reported by several groups that all diabetic patients with HNF1β mutations exhibit renal dysfunction (43, 49, 50). These research results imply that HNF1β plays a more dominant role in the kidney than HNF1α.

We found that HNF1α SNPs related to glycosuria were mainly concentrated in the dimer domain and DNA binding domain of HNF1α (**Table 3**), indicating that HNF1α regulates the transcription of genes that control the renal glucose threshold along with other transcription factors, likely by forming a dimer with HNF1β to regulate the genes involved in glucose transport or reabsorption in the kidney. However, this hypothesis is only our speculation and needs to be further confirmed *via* by experiments.

**Table 3:** The HNF1A SNPs associated glycosuria.

Location		Nucleotide Change at DNA Level	Mutation	Age of Onset of the Subject (Yrs)	Sex	IBM	Ref
Genomical DNA	Codon						
Exon 1	31	c.92G>A	p.G31D	15	Male	15.9	(51)
Exon 1	55,56	c.161-165delGAGGG	R55G56fsdelGAGGG	17	Male	24.6	(46)
Exon 1	98	c.283C>T	p.A98V	5	Male	NA	(52)
Exon 2	142	c.425C>T	p.S142F	9	Female	27.2	(46)
Exon 2	171	c.511C>T	p.R171X	14	Female	20.9	(46)
Exon 3	224	c.670C>T	p.P224S	NA	Male	NA	(44)
Exon 3	230-236	c.687_707del	p.E230_C236del	13	Female	22	(53)
Exon 4	272	c.815G>A	p.R272H	NA	Male	NA	(54)
Intron 5	Splice site	c.955+2 T>A	IVS5nt + 2T→A	20	Male	24.1	(46)
Exon 4	271	c.811C>T*	p.R271W	15	Female	NA	(55)
Intron 7	Splice site	c.1502-6G>A*	IVS7nt-6G>A	17	Female	NA	(55)

*This SNP was related to Renal malformations.

NA, Not available.

#### Cardiovascular System

HNF1A-MODY is a type of nonketotic diabetes characterized by progressive hyperglycemia in childhood, adolescence, and early adulthood and has a high risk of chronic microvascular complications (56, 57). The plasma glucose of patients deteriorates with age, and the risk of microvascular complications increases. In addition to the cardiovascular burden caused by uncontrolled blood glucose, some studies have found that some SNPs can increase the risk of vascular and cardiac diseases, such as I27L, A98V, and S487N (57). Some SNPs are closely related to dyslipidemia, a significant independent risk factor for cardiovascular abnormalities (58). This association may be related to the involvement of HNF1α in the synthesis of liver-related lipoproteins and liver lipid metabolism.

Some researchers reported that the incidence of cardiovascular and microvascular complications is similar to that in patients with T1D and T2D and is associated with poor glycemic control (25, 56, 59). However, Babaya N, et al. reported that the concentration of high-density lipoprotein cholesterol (HDL-C) in HNF1A-MODY (I27L carrier) is higher than that in normal individuals (60). HDL-C can reduce cardiovascular risk; thus, the incidence rate of coronary heart disease in HNF1A-MODY patients with I27L carrier may be lower than that in T1D patients and T2D patients.

#### Cancer

Recent studies showed that MODY3 is a risk factor for pancreatic cancer (61, 62). HNF1α gene mutation is related to pancreatic, liver, and kidney tumors (63–66). It has been reported that HNF1α mutations (p.E32 * and p.L214Q) are related to hepatocellular tumors (67). Somatic HNF1α mutations are found in approximately 1% to 2% of hepatocellular carcinomas and usually occur in adenomas. HNF1α mutations increase the risk of the malignant transformation of hepatocellular adenomas (67).

### Treatment

HNF1A-MODY3 can cause severe diabetic retinopathy, diabetic nephropathy, diabetic peripheral neuropathy, and other complications (59). Therefore, early diagnosis and timely treatment are very important for blood glucose control, delaying the occurrence and development of complications and improving the quality of life.

The phenotype of HNF1A-MODY is characterized by mild nonprogressive hyperglycemia, progressive hyperglycemia, and hyperglycemia with extra-pancreatic characteristics (25, 26). In patients diagnosed with mild hyperglycemia, diet seems to be a reasonable and effective treatment strategy; however, in the case of progressive hyperglycemia, pharmacological methods should be attempted (68, 69). The treatment of HNF1A-MODY patients depends on their age and HbA1c level (64). Patients with HNF1α mutations are very sensitive to the oral hypoglycemic drug sulfonylurea (70, 71). It is speculated that this may be due to the decreased liver clearance of some sulfonylurea derivatives in patients with HNF1α gene mutations, resulting in an increase in serum levels (72). The increased circulating levels of these drugs could explain the enhanced efficacy. The response of HNF1A-MODY patients to sulfonylureas is five times higher than that to standard metformin (73). However, in T2D, the efficacy of the two drugs has been demonstrated to be almost the same (73). Sulfonylureas usually control blood glucose better than insulin therapy in patients with HNF1A-MODY, and the fasting hypoglycemic effect is also good (70). HNF1A-MODY patients show obvious sensitivity to oral sulfonylurea drugs. The failure of sulfonylurea treatment is rare and occurs in only a few patients with the c.618G>A mutation (74). Therefore, low-dose sulfonylurea drugs (such as 20-40 mg/day gliclazide) are preferred for long-term treatment and should be regarded as the first-line treatment for HNF1A-MODY (73).

However, studies have shown that patients with some variants no longer respond to the above treatments. Patients with p.His126Asp do not respond to sulfonylureas (low and high doses), dipeptidyl peptidase-4 inhibitors (DPP-4 inhibitors), or glucagon-like peptide-1 receptor agonists (GLP-1Raa), also known as incretin analogs (75). Low-dose sulfonylurea therapy is the first-line therapy for MODY3 but does not show special sensitivity to HNF1α-related T2D (76). For example, Mexican carriers of the HNF1α p.E508K variant have no increased sensitivity to sulfonylureas (76).

In a word, HNF1A-MODY is a disease with genetic and clinical heterogeneity. However, MODY3 still has strong common characteristics. Patients with HNF1A-MODY usually have low high-sensitivity C-reactive protein (hsCRP) levels (77, 78). In addition, although MODY3 is a type of monogenic diabetes, the severity can differ with different genetic backgrounds and environmental factors. Previously, MODY3 patients were generally not overweight or obese and lacked other risk factors for T2D, such as hypertension or dyslipidemia (79). However, as an increasing number of people have become overweight or obese, individuals with MODY3 may also be overweight or obese. According to statistics, nearly 30% of HNF1A-MODY patients in the United States are overweight or obese, rendering the differential diagnosis between HNF1A-MODY3 and T2D more difficult (80). Therefore, although some clinical guidelines for MODY3 diagnosis exist, HNF1A-MODY3 patients often fail to meet all diagnostic criteria or are misdiagnosed with T1D or T2D.

## Association Between HNF1A Polymorphism and T2D

Studies have found that some HNF1α SNPs do not cause MODY3 but increase the susceptibility to T2D (**Supplementary Table 1**). A large-scale association analysis of a group of people mainly of European ancestry showed that the T2D susceptibility loci existed near HNF1α (81). Moreover, SNPs related to T2D are closely related to race. The p.E508K variant was found only in T2D patients in Mexico (82). The rare p.A98V allele may be associated with T2D in the Caucasian population (83). However, the p.A98V allele does not appear to be associated with T2D in Asian populations (84). Among them, the most famous locus is G319S. The G319S polymorphism of HNF1α is positively correlated with the high prevalence of T2D in Canadian Aborigines (85, 86).

Gene sequencing revealed that the G319S mutation in HNF1α was associated with an increased incidence rate of T2D in the Oji Cree ethnic group in Canada. The specific and positive predictive values of G319S carriers suffering from T2D before the age of 50 were 97% and 95%, respectively (87). G319S is associated with a distinct form of T2D characterized by onset at an earlier age, higher postprandial plasma glucose, and lower body mass index (BMI) (85). In patients with T2D, compared to those with G319/G319, the BMIs of individuals with S319/S319 and S319/G319 were significantly lower, and postprandial blood glucose was significantly higher (85). In nondiabetic individuals, the plasma insulin of S319/G319 heterozygotes was significantly lower than that of G319/G319 homozygotes (85). A lower BMI coupled with the decrease of insulin secretion before the onset of diabetes is the pre-diabetic physiologic state of individuals with HNF1A-T2D. This group is different from those with other types of T2D caused by other genes. The latter group is often generally obese and has a high BMI with insulin resistance before obvious diabetes. Moreover, smoking appears to increase the risk of diabetes in HNF1α G319S carriers (88). G319s is located in HNF1α transactivation sites, which are rich in proline II domains (**Figure 1**), with changes in conserved glycine residues. The function of the protein carrying the G319S mutation was found to be impaired *in vitro* (87), and the transcription ability was reduced by approximately 50% (88). However, this mutation did not affect DNA binding or protein stability. There is no evidence that the mutant protein has a dominant-negative effect. The G319S mutation affected the transcriptional shear of HNF1α. Two abnormal transcripts and an alteration in the relative balance of normal splicing products were produced by the G319S variant (89). Two abnormal transcripts present only in the G319S cells included premature termination codons resulting from the inclusion of seven nucleotides from intron 4 or the deletion of exon 8. A novel isoform lacking the terminal 12 bases of exon 4 was increased compared with that in control cell lines and human pancreatic tissue. The combination of the reduced activity of the G319S protein and abnormal splicing transcripts may increase the susceptibility to diabetes.

HNF1A-SNPs associated with T2D only increase the risk of T2D. Obvious T2D requires other factors, such as genetic and/or environmental factors. An interesting example can be found in stories of a new HNF1α variant c.539C >T (p.Ala180Val) in two families in Norway (90). There was an obvious difference in the probability of T2D between these two families (90). p.Ala180Val is a mutation that affects highly conserved amino acid residues in proteins. The HNF1α mutant p. Ala180Val does not cause MODY3 but may increase the risk of T2D. This variation was found to be completely separate from diabetes in one family (family A), but the data did not support its role as a pathogenic factor of MODY3. In the other family (family B), there was no clear genotype/phenotype correlation. Two diabetic patients and one individual with normal plasma glucose levels in this family were homozygous mutation carriers. In family A, the mutation carriers had similar metabolic syndromes, including obesity, diabetes, hypertension, and dyslipidemia. Moreover, the nonmutation carriers in the family were overweight or obese, although they had normal blood glucose levels, but they were as overweight or obese as the family members carrying the mutation. Genetic factors may be related to the metabolic abnormalities in family A. Therefore, it can be speculated that HNF1α p.Ala180Val could lead to a certain degree of β cell dysfunction; however, it does not cause significant glycemic abnormalities. The genetic background of family A associated with metabolic abnormalities increased insulin resistance in the HNF1α p.Ala180Val carriers, leading to marked diabetes. In contrast, family B members did not present with obesity or metabolic syndrome, but some female mutation carriers had a history of GDM. Therefore, we can speculate that p.Ala180Val can increase the susceptibility to hyperglycemia but cannot lead to obvious diabetes. Under the influence of additional stress, such as other genetic factors responsible for obesity or pregnancy, p.Ala180Val carriers develop peripheral insulin resistance or permanent impairment in β cellular function, resulting in marked diabetes. The difference between the two families may be due to the accumulation of the higher-risk variant for T2D in family A. Overall, lifestyle and environmental factors may also play a role in the phenotypic differences.

Low-dose sulfonylurea therapy is the first-line therapy for MODY3 but does not show special sensitivity to T2D. For carriers of HNF1α variants that may cause T2D, dietary treatment is the first recommendation.

## Association Between HNF1A Polymorphism and GDM

GDM is a common complication of pregnancy that has adverse effects on the short-term and long-term health of women and their children (91). Approximately 2-5% of pregnant women develop GDM during pregnancy, and the incidence rate has significantly increased over the past 10 years (92). GDM is diagnosed when any degree of glucose intolerance occurs during pregnancy for the first time. GDM is also heterogeneous diabetes, with varying degrees of diabetes caused by β cell dysfunction (92). When pancreatic β cells can no longer compensate for the increased insulin resistance during pregnancy, they show varying degrees of glucose intolerance. Although the pathogenesis of the disease is still largely unknown, GDM is considered the result of an interaction between genetic and environmental risk factors. Age, obesity, and a high-fat diet are some important nongenetic factors (93).

Many studies have found that HNF1α SNP increases the risk of GDM. HNF1α is one of the pathogenic genes of GDM in Danish women. The sequencing of 354 Danish GDM patients revealed five diabetes-susceptible variants of HNF1α in seven HNF1α mutation carriers (94). Only those with the Gly288fs* variant were diagnosed with diabetes before the follow-up period and received insulin treatment. The remaining HNF1α mutation carriers were not diagnosed with diabetes before the follow-up period. p.A98V was associated with significant impairment of serum insulin and C-peptide secretion during an oral glucose tolerance test in previously GDM-free glucose-tolerant women (95). The p.I27L polymorphism of HNF1α seems to increase the risk of GDM in Scandinavian women (96). The p.I27L gene was found to increase GDM by increasing insulin resistance in Turkish women (97). In Scandinavian women, the p.I27L polymorphism of HNF1α also increased the risk of GDM (96). The p.I27L TT genotype was associated with an increased risk of preeclampsia in patients with GDM by increasing blood pressure and urinary protein (97). No difference in weight was observed compared to non-diabetic pregnant women with HNF1α mutation during the entire pregnancy.

Pregnant women with HNF1α mutation may develop GDM due to islet dysfunction. Pregnancy is a significant source of stress for women, which leads to the increase of insulin demand. If the need for insulin cannot be met, GDM will gradually develop. Dyslipidemia during pregnancy increases the risk of pregnancy complications. The lipid profile has a strong genetic determinant. In 2017, Xiaojing Wang et al. found that the total cholesterol levels of pregnant women carrying the T alleles of rs1169309 in the HNF1α gene were elevated, which could significantly increase the risk of GDM (98). Insulin resistance may also be involved in the occurrence of GDM.

Compared with other gene mutation carriers, GDM HNF1α mutation carriers exhibit a significant reduction in hsCRP expression (94). hsCRP is encoded by the CRP gene, which has an HNF1α transcription factor-specific binding site (65, 99). SNPs in HNF1α have been associated with CRP levels in different populations (100, 101). The expression of hsCRP in GDM patients is higher than that in HNF1A-MODY patients (84). This finding indicates that GDM caused by HNF1α results in the same susceptibility to diabetes as the T2D variant, but insufficient penetrance leads to clinical MODY.

Dyslipidemia in pregnancy increases the risk of pregnancy complications. Therefore, pregnant women who are HNF1α gene mutation carriers should pay special attention to their health management and consume a reasonable diet during pregnancy. If HNF1α mutation carriers have hyperglycemia during pregnancy, they should not be treated with sulfonylureas and need to be treated with insulin.

## Mechanism of Abnormal Blood Glucose Associated With HNF1A Gene Polymorphism

HNF1α is expressed in embryonic development, infancy, and adulthood. Moreover, the expression distribution has strong tissue specificity, mainly concentrated in the tissues responsible for metabolism, such as the pancreas and liver. Through transcriptomics and antibody-based proteomics, the analysis of human tissue-specific expression showed that the expression level of HNF1α varies in human tissue and controls the transcriptional expression of many genes in the tissue (102). According to incomplete statistics, HNF1α can bind at least 106 target genes in the pancreas (103), which may explain why the mutation location of the HNF1α gene determines the age of diabetes onset. The above results indicated that HNF1α may play varied and important roles in the tissues. Below, we focus on the possible mechanism of HNF1α for glucose homeostasis in the pancreas and liver.

### Functions in Pancreas

#### Maintain the Mature β Cell Function

The dysfunction of mature β cells is the main reason for the hyperglycemia caused by HNF1α. Basal insulin secretion and insulin production corresponding to high glucose stimuli are the basic functions of mature β cells. MODY3 patients have insulin secretion disorder, and the islet secretion function gradually declines as the disease worsens (104). Fasting insulin and glucose-stimulated insulin secretion were found to be abnormal in 40 HNF1α mutation carries from Britain and France (105). The insulin sensitivity was elevated in these individuals as well as the proinsulin to insulin ratio. The results indicated that HNF1α mutation directly altered basal insulin secretion, rather than glucose sensing insulin secretion. However, a recent islet study of a 33-year-old patient with MODY3, who was misdiagnosed with T1D for 17 years, found that HNF1α causes insulin deficiency diabetes by affecting glucose-stimulated insulin secretion (106). The two results were different, possibly due to different HNF1α variants. In addition, G319S carriers reportedly showed a decrease in insulin secretion before diabetes appeared (85). This indicated islet dysfunction also exists in HNF1A-T2D patients. Therefore, we think HNF1α plays very important and different roles in basal insulin secretion and insulin production corresponding to high glucose stimuli. In order to better study the role of HNF1α in β cell dysfunction, it is necessary to compare the islet function of HNF1α mutants with different degrees of clinical severity to identify the domain of HNF1α involved in the secretion of basic insulin and the domain involved in the secretion of insulin stimulated by high glucose.

HNF1α can regulate many genes involved in insulin secretion (16, 42) and directly bind to the promoter region of the insulin gene, positively regulating the transactivation of the latter. In addition to directly regulating insulin transcription, HNF1α was found to directly regulate the GLUT2 and HNF4 pyruvate kinase genes, which are the key genes involved in cellular insulin secretion (103). In addition, heterozygous HNF1α variations change the expression of key enzymes involved in mitochondrial glucose metabolism (107). In conclusion, HNF1α is an important transcription factor for the maintenance of β cell function, but the specific mechanism needs to be further studied.

#### Development and Maturation of Islets

HNF1α is involved in the development and maturation of islets. In normal embryonic mouse pancreas, the expression of HNF1α was detected on embryonic day 10.5 (E10.5) (108). When the dominant negative p291fsinsc HNF1α mutation is specifically expressed in β cells (driven by the rat Ins2 promoter), the islets are gradually disordered with reduced cells numbers, and the cells are dispersed in the islet (109, 110). Signs of serious cell damage can be observed, including vacuolization, immature secretory granules, swollen mitochondria, and expanded endoplasmic reticulum (109, 110). Nkx6.1 is a homologous domain transcription factor that plays a role in pancreatic development and the maintenance of mature β cellular function (111). Studies have shown that Nkx6 can directly activate the expression of HNF1α (112). HNF1α in turn directly activates the expression of MafA, which encodes transcription factors produced later in the developing pancreatic transcription program and is expressed only in differentiated insulin hormone positive cells (113).

Some studies indicated that HNF1α is not limited to β cell development and may also affect α cell development. HNF1α whole-body knockout mice died at 6 weeks after birth with small islets and a high α/β ratio (114). This phenomenon is consistent with the islet results for diabetic patients with HNF1α mutation (106). The increased α/β cell ratio may be caused by the slightly higher quality of α cells in the pancreas of these patients. The manifestations of cell hyperfunction, excessive glucagon secretion, weakened negative feedback to glucose, and decreased intestinal glucagon effect are observed in MODY3 patients (115). In 2020, Kazuya Yamagata et al. found HNF1α can inhibit glucagon secretion by regulating SGLT1 expression in α cells (116). HNF1α was found to inhibit α cell characteristics in modeling monogenic diabetes using human embryonic stem cells through mutations in HNF1α (117).

Although further research is needed, it is clear that HNF1α plays a role in pancreatic organogenesis, endocrine and exocrine cell differentiation, and growth by influencing islet specific transcription factors.

### Function in Liver

In the liver, HNF1α may affect the balance of blood glucose through regulated glucose and lipid metabolism. HNF1α directly binds to the promoter region of the glucose 6-phosphate transporter (G6PT) gene, the key enzyme of the glucose-6-phosphatase (G6Pase) system, to promote the transcription of the latter (118). Compared with HNF1α (+/+) and HNF1α (+/-) littermates, hepatic G6PT mRNA levels and microsomal G6P transport activity are markedly reduced in HNF1α (-/-) mice (118). The G6Pase system is essential for the maintenance of glucose homeostasis. Thus the HNF1α variant may cause abnormal glucose homeostasis through the G6Pase system. After HNF1α deletion in the liver, the expression of genes encoding fatty acid synthetic enzymes (fatty acid synthase and acyl-CoA carboxylase) and peroxisomal β-oxidation enzymes (CYP4A3, bifunctional enzyme, and thiolase) increased (119). However, the expression of the hepatic fatty acid binding protein (L-FABP) gene decreased significantly (119). Two HNF1α binding sites were found in the 5’ promoter region of L-FABP by sequence analysis. Cell experiments confirmed that HNF1α was necessary for the transactivation of L-FABP (119). We speculated that HNF1α mutation may disrupt the balance of the blood glucose through regulating glucose metabolism and adipogenesis.

HNF1α also participates in the transcription of apolipoprotein genes (120, 121). The HNF1α G319S genotype was significantly correlated with the total plasma cholesterol, low-density lipoprotein cholesterol (LDL-C), and apolipoprotein (Apo) B concentration in Oji Cree individuals with T2D (122). In Oji Cree people who did not have T2D, we found that the HNF1α G319S genotype was significantly associated with the plasma concentrations of HDL-C and apolipoprotein AI (123). The phenotype was not related to plasma triglyceride or lipoprotein (a). I27L had a protective effect on hypertriglyceridemia in these individual samples (57). The above studies all indicated that HNF1α plays a role in the lipid profile of diabetic individuals. These data enhance our understanding of the complex interactions among genes, hyperglycemia, and cardiovascular risk factors in T2D. Some studies have shown that a decreased expression of HNF1α increases the risk of fatty liver, which is closely related to insulin resistance (122, 124).

In summary, HNF1α SNPs responsible for the abnormality of blood glucose may be caused by changes in the development, promotion, and death of β cells, the maintenance of mature pancreatic function, as well as glucose and lipid metabolism in the liver.

## Relationship Between HNF1A and Different Types of Diabetes

HNF1A-MODY3 is characterized by not only gene heterogeneity but also phenotype heterogeneity. For example, the two human variants HNF1α (P408H) and HNF1α (P409H) can be considered different variants (125, 126). A possible reason is that these two adjacent sites regulate the promoters of two different HNF1A-targeted genes. This is not surprising and can be explained by the interactions between HNF1α and different coactivators, which are necessary for the complete activation of different HNF1α downstream target promoters (127). The above phenomena show that HNF1α plays many roles and is widely involved in the strict and fine regulation of many genes responsible for metabolism.

Some HNF1α SNPs cause obvious MODY3, while others do not cause MODY3 but increase the risk of T2D or GDM. These are very interesting phenomena. By determining the underlying mechanism of these phenomena, we may improve our ability to individualize diabetes treatment. First, what is the difference between MODY, T2D, and GDM? The biggest difference is the age/time of onset. The onset age of MODY is below 25 years old, while T2D occurs in adulthood, and the onset of GDM is obvious diabetes in the middle and later stages of pregnancy. The earlier the onset, the more dominant is the role of genetic factors in the occurrence of diseases. The impact of environmental and other physical factors is greater with later onset. In essence, pregnancy is a stressor on the female body. The second major difference is whether the family history is obvious. The more obvious the family history is, the more important is the role of genetic factors. HNF1α heterozygous mutations lead to MODY3. Since an HNF1α allele is normal in MODY patients, it can be deduced that the expression level of HNF1α is important in this group. MODY plays a vital role in cell function, especially in β cells. A lack of sufficient protein levels leads to a significant loss of function of mutant alleles (104), and dominant negative effects caused by interference between mutant products and wild-type forms lead to the formation of inactive heterodimers (128). HNF1A-MODY-related variants function through one of the above mechanisms, i.e., a simple loss of function or a dominant-negative mechanism. T2D and GDM are diabetes types caused by multiple factors. The HNF1α SNP, which triggers T2D and GDM, has a certain impact on the process of abnormal blood glucose. Therefore, we speculate that HNF1α SNPs related to T2D or GDM partially impair the function of the HNF1α protein.

The HNF1α protein contains the following three functional domains: an N-terminal dimer domain, a DNA binding region containing a nuclear localization signal, and a C-terminal transactivation domain (**Figure 1**). The N-terminal dimeric domain (residues 1-32) forms a four-helix bundle, two of which separate the α-helix in a circle to form a dimer (129, 130). Usually, two HNF1αs form a homodimer or one HNF1α associates with the HNF1β transcription factor, which has a similar structure, to form a heterodimer (47). The DNA binding domain (DBD) of HNF1α binds reverse palindrome 5’-gttaatnataac-3’ and forms a helix-to-helix structure (131). The DBD includes two POU subdomains, i.e., POU-specific domain (POUs, amino acids 91-181) and POU-homologous domain (POUH, amino acids 203-279) (104). The amino acid positions of DBDs differ slightly. POUs are an integral part of HNF1α and play a vital role in maintaining protein stability (132). Comparing the common HNF1α SNPs that cause MODY3 with those that cause T2D, most of the former are concentrated in exon 1, 2, and 4, which encode the DNA binding region of the C-terminal transactivation domain of HNF1α (**Figure 1**). The HNF1α SNP leading to T2D is concentrated in exons 8 and 9. The above sites of the HNF1α SNP that lead to T2D are almost outside the proline rich activation domain II of HNF1α, which is located in the transactivation domain.

SNP mutations may affect the function of the HNF1α protein through the following mechanisms: 1) Affecting the ability of DNA to bind the transcriptional regulatory region of the target gene; 2) Affecting the transcriptional activity of HNF1α; 3) Affecting the nuclear entry ability of the HNF1α protein; 4) Affecting the stability of the HNF1α protein; and 5) Affecting the expression of the HNF1α protein, especially SNPs located in the promoter region (107, 125, 133). In 2017, Najmi et al. found that the transcriptional activity or DNA binding ability of HNF1α variants that cause T2D was between those of normal wild-type protein and the HNF1A-MODY variant (134). The above interesting study may indicate that HNF1α participates in multiple signaling pathways involved in abnormal blood glucose and that the HNF1α variants identified among T2D patients may lack sufficient penetrance to drive diabetes but still increase the susceptibility to diabetes.

In summary, the HNF1α gene is highly polymorphic, and the clinical phenotypes caused by different SNPs may vary greatly. Therefore, to better manage the abnormal blood glucose levels caused by HNF1α mutations, genotype identification should be performed to obtain detailed information concerning HNF1α.

## Conclusion and Future Perspectives

To date, many HNF1α SNPs have been identified and are widely distributed in the HNF1α gene. HNF1α-associated diabetes mellitus has larger clinical heterogeneity. Significant differences have been found in abnormal plasma glucose caused by SNPs at different sites. Patients with some variants do not have diabetes throughout their lives, some with other variants show serious hyperglycemia in childhood, while others show hyperglycemia in their older years. HNF1α is a tissue-specific transcription factor mainly expressed in the pancreas and liver. Hundreds of target genes have been found in these tissues. In the pancreas, HNF1α not only maintains the function of mature pancreatic β cells, but also affects the development and maturation of β cells. In the liver, HNF1α abnormality inhibits hepatic glycogen decomposition and promotes lipolysis. Uncovering the mechanism underlying why different HNF1α mutations cause different types of diabetes will provide a theoretical basis for personalized prevention and treatment of HNF1α-associated hyperglycemia and will also benefit research on T2D, which is the main type of diabetes. HNF1α may cause different forms of diabetes due to different levels of penetrance and genetic backgrounds. These differences have a certain correlation with the location of the SNPs. The SNP for HNF1A-MODY is often located in the DNA binding region, while those for T2D and GDM are located in a different region. Functional studies have shown that the transcriptional activity or DNA binding ability of the HNF1α variant of T2D is between those of the normal wild-type protein and the HNF1A-MODY variant. This finding may indicate that the HNF1α variants identified among T2D patients may lack sufficient penetrance to drive diabetes but still increase the susceptibility to diabetes. Accordingly, there must be differences in the treatment of diabetes caused by different SNPs. Low-dose sulfonylurea therapy is the first-line therapy for MODY3 but does not show special sensitivity to T2D. For carriers of the HNF1α variants that may cause T2D or GDM, dietary treatment is the first recommendation. We hope research on the pathogenesis and drug treatments for HNF1A-T2D and GDM will progress in the near future with studies on the function of HNF1α.

## Author Contributions

L-ML and B-GJ collected information. L-LS and L-ML wrote the manuscript. All authors contributed to the article and approved the submitted version.

## Funding

This study was supported by grants from the National Natural Science Foundation of China (81672721), National Key Research and Development Projection (No. 2018YFC1314100), Shanghai Science Foundation (17DZ1910604 and 19ZR1456900), Diabetes Talent Research Project of China International Medical Foundation (2018-N-01-26), Key Laboratory Development Project of Minimally Invasive Techniques & Rapid Rehabilitation of Digestive System Tumor (21SZDSYS05).

## Conflict of Interest

The authors declare that the research was conducted in the absence of any commercial or financial relationships that could be construed as a potential conflict of interest.

## Publisher’s Note

All claims expressed in this article are solely those of the authors and do not necessarily represent those of their affiliated organizations, or those of the publisher, the editors and the reviewers. Any product that may be evaluated in this article, or claim that may be made by its manufacturer, is not guaranteed or endorsed by the publisher.
