# Supplementary material for: HNF1A：From Monogenic Diabetes to Type 2 Diabetes and Gestational Diabetes Mellitus
Source: Front Endocrinol (Lausanne). 2022 Mar 1;13:829565. doi: 10.3389/fendo.2022.829565 (PMC8921476; doi:10.3389/fendo.2022.829565)
Supplement: Supplementary file 1 [file DataSheet_1.pdf]

Supplementary Material

Supplementary Tables

Supplementary Table 1. The HNF1A SNPs associated with MODY3 or T2D.

**Location**				
**Exon/Intron**	**Codon**	**Nucleotide and systematic name**	**Amino acid change**	**Diabetes type**
Promoter		c.-538G>C	{-538(G-C)}	MODY3
Promoter		c.-373C>T	{-373(C-T)}	MODY3
Promoter		c.-283A>C	{-283(A-C)}	MODY3
Promoter		c.-218T>C	{-218(T-C)}	MODY3
Promoter		c.-158insGGGTTGG	{-insGGGTTGG}	MODY3
Promoter		c.-58A>C	{-58(A-C)}	MODY3
Promoter		c.-58A>C	{-58(A-C)}	MODY3
Promoter		c.102G>C	{102(G-C)}	MODY3
Exon 1	6	c.16A>C	p.S6R	MODY3
Exon 1	7	c.19C>T	p.Q7X	MODY3
Exon 1	12	c.35T>A	p.L12H	MODY3
Exon 1	17	c.51C>G	p.L17L	MODY3
Exon 1	20	c.59G>C	p.G20A	MODY3
Exon 1	27	c.79A>C	p.I27L	MODY3
Exon 1	31	c.91G>T	p.G31X	MODY3
Exon 1	41	c.121G>T	p.E41X	MODY3
Exon 1	47	c.140G>A	p.G47E	MODY3
Exon 1	48	c.142C>A	p.E48K	MODY3
Exon 1	54	c.160C>T	p.R54X	MODY3
Exon 1	55/56	c.161-165 delGAGGG	R55G56fsdelGAGGG	MODY3
Exon 1	58	c.174T>G	p.A58A	MODY3
Exon 1	74	c.222G>T	p.T74T	MODY3
Exon 1	80	c.240C>G	p.D80E	MODY3
Exon 1	98	c.293C>T	p.A98V	MODY3
Exon 1	103	c.307G>A	p.V103M	MODY3
Exon 1	107	c.319C>G	p.L107I	MODY3
Exon 2	112	c.335C>T	p.P112L	MODY3
Exon 2	114	c.340C>T	p.R114C	MODY3
Exon 2	122	c.365A>G	p.Y122C	MODY3
Exon 2	128	c.383T>C	p.I128N	MODY3
Exon 2	129	c.385C>A	p.P129T	MODY3
Exon 2	130	c.388C>T	p.Q130X	MODY3
Exon 2	131	c.391C>T	p.R131W	MODY3

Exon 2	131	c.392G>A	p.R131Q	MODY3
Exon 2	133	c.397G>A	p.V133M	MODY3
Exon 2	134	c.402C>T	p.V134V	MODY3
Exon 2	142	c.425C>T	p.S142F	MODY3
Exon 2	142	c.426delG	S142fsdelG	MODY3
Exon 2	143	c.427C>T	p.H143Y	MODY3
Exon 2	159	c.476G>A	p.R159W	MODY3
Exon 2	159	c.476G>A	p.R159Q	MODY3
Exon 2	162	c.485T>C	p.L162P	MODY3
Exon 2	171	c.511C>G	p.R171G	MODY3
Exon 2	171	c.511C>T	p.R171X	MODY3
Intron 2		c.527-1G>A	IVS2nt-1G-->A	MODY3
Exon 3	196	c.587_590delCCAA	T196fsdelCCAA	MODY3
Exon 3	203	c.607C>G	p.R203G	MODY3
Exon 3	229	c.686G>A	p.R229Q	MODY3
Exon 3	235	c.703G>C	p.E235Q	MODY3
Intron 3	Intron	c.714-1G>A	IVS3－1G>A	MODY3
Exon 4	241	c.721T>	p.C241R	MODY3
Exon 4	241	c.721T>G	p.C241G	MODY3
Exon 4	245	c.733G>C	p.G245R	MODY3
Exon 4	260	c.779C>T	p.T260M	MODY3
Exon 4	263	c.788G>A	p.R263H	MODY3
Exon 4	269	c.805G>C	p.A269P	MODY3
Exon 4	271	c.812G>	p.R271G	MODY3
Exon 4	271	c.812G>A	p.R271Q	MODY3
Exon 4	271	c.811C>T	p.R271W	MODY3
Exon 4	272	c.814C>	p.R272H	MODY3
Exon 4	272	c.814C>T	p.R272C	MODY3
Exon 4	276	c.827C>A	p.A276D	MODY3
Exon 4	288	c.864G>C	p.G288G	MODY3
Exon 4	290	c.869delC	Pro290fsdelC	MODY3
Exon 4	291	c.872delC	Pro291fsdelC	MODY3
Exon 4	291	c.873delA	Pro291fsdelA	MODY3
Exon 4	291	c.873delG	Pro291fsdelG	MODY3
Exon 4	291	c.873Insertion C	Pro291fsinsC	MODY3
Exon 4	291	c.872dupC	Pro291dupC	MODY3
Exon 4	291	c.872^83insC	Pro291fsinsC	MODY3
Exon 4	292	c.878delG	Pro292fsdelG	MODY3
Exon 4	301	c.901G>A	p.A301T	MODY3
Intron 4		c.956-2 A>G	c.956-2 A>G	MODY3
Exon 5	321	c.962 G>A	p.R321H	MODY3

Exon 5	349	c.1047C>T	p.H349Q	MODY3
Exon 5	355	c.1064C>A	p.S355X	MODY3
Exon 6	379	c.1136^1137insC	P379fsinsC	MODY3
Exon 6	379	c.1136-1137delT	P379fsdelT	MODY3
Exon 6	379	c.1136-1137delCT	P379fsdelCT	MODY3
Exon 6	398	c.1194T>C	p.Q398X	MODY3
Exon 6	414	c.1238_1239dupCC	p.I414fs	MODY3
Exon 6	432	c.1295C>G	p.S432C	MODY3
Exon 6	432	c.1295C>A	p.S432Y	MODY3
Exon 7	443	c.1328delCA	A443fsdelCA	MODY3
Exon 7	445	c.1333_1334delAG	S445fsdelAG	MODY3
Exon 7	447	c.1340C>T	p.P447L	MODY3
Exon 7	447	c.1340C>G	p.P447L	MODY3
Exon 7	459	c.1375C>T	p.L459L	MODY3
Exon 7	466	c.1396C>T	p.Q446*	MODY3
Exon 7	473	c.1417C>T	p.Q473X	MODY3
Exon 7	487	c.1460G>A	p.S487N	MODY3
Exon 7	488	c.1463delC	P488fsdelC	MODY3
Exon 7	495	c.1483C>T	p.Q495X	MODY3
IVS 7	Splice site	c.1501+7A>G	c.1501+7A>G	MODY3
Exon 8	519	c.1556C>T	p.P519L	MODY3
Exon 8	521	c.1562C>T	p.T521I	MODY3
Exon 8	531	c.1592G>C	p.S531T	MODY3
Exon 9	547/548	c.1641-1642delTG	T547E548fsdelTG	MODY3
Exon 9	559	c.1677^1678insA	A559fsinsA	MODY3
Exon 9	584	c.1752^1753insTC	L584S585fsinsTC	MODY3
Exon 9	588	c.1764-1765insGCCA	P588fs-insGCCA	MODY3
Exon 9	588	c.1764delAC	P588fs-delAC	MODY3
Intron 9	Splice site	c.1768+1 G>A	c.1768+1 G>A	MODY3
Exon 10	617	c.1849 G>A	p.V617I	MODY3
Exon 10	618	c.1854 C>G	p.I618M	MODY3
Exon 10	620	c.1859 C>T	p.T620I	MODY3
Exon 1	31	c.92G>A	p.G31D	T2D
Exon 1	48	c.142G>A	p.E48K	T2D
Exon 1	62	c.185A>G	p.N62S	T2D
Exon 1	97	c.290C>T	p.A97V	T2D
Exon 1	100	c.298C>A	p.Q100K	T2D
Exon 2	114	c.341G>A	p.R114H	T2D
Exon 2	131	c.392G>A	p.R131Q	T2D
Exon 2	141	c.422_423InsT	p.Q141Hfs *47	T2D
Exon 3	180	c.539C>T	p.Ala180Val	T2D
Exon 3	191	c.572G>A	p.G191D	T2D
Exon 3	196	c.586A>G	p.T196A	T2D
Exon 4	254	c.760C>A	p.L254M	T2D
Exon 4	263	c.787C>T	p.R263C	T2D

Exon 4	275	c.825del	p.E275del	T2D
Exon 4	319	c.955G>A*	p.G319S	T2D
Exon 5	322	c.965A>G	p.Y322C	T2D
Exon 6	389	c.1165T>G	p.L389V	T2D
Exon 7	469	c.1405C>T	p.H469Y	T2D
Exon 7	487	c.1460G>A	p.S487N	T2D
Exon 8	505	c.1513C>A	p.H505N	T2D
Exon 8	508	c.1522G>A#	p.E508K	T2D
Exon 8	514	c.1541A>G	p.H514R	T2D
Exon 8	515	c.1544C>A	p.T515K	T2D
Exon 8	537	c.1610C>G	p.T537R	T2D
Exon 9	566	c.1696C>A	p.H566N	T2D
Exon 9	574	c.1720G>A	p.G574S	T2D
Exon 9	577	c.1729C>G	p.H577D	T2D
Exon 9	583	c.1748G>A	p.R583Q	T2D
Exon 10	619	c.1856G>A	p.E619K	T2D
